# Supplementary material for: The clinical-stage drug BTZ-043 accumulates in murine tuberculosis lesions and efficiently acts against Mycobacterium tuberculosis
Source: Nat Commun. 2025 Jan 18;16:826. doi: 10.1038/s41467-025-56146-9 (PMC11742723; doi:10.1038/s41467-025-56146-9)
Supplement: Supplementary file 1 — Supplementary Information [file 41467_2025_56146_MOESM1_ESM.pdf]

Supplementary Information

The clinical-stage drug BTZ-043 accumulates in murine tuberculosis lesions and efficiently acts against *Mycobacterium tuberculosis*

Andreas Römpp[1,2,*], Axel Treu[1,2,‡], Julia Kokesch-Himmelreich[1,2,‡], Franziska Marwitz[3,4,‡], Julia Dreisbach[2,5], Nadine Aboutara[3,4], Doris Hillemann[6], Moritz Garrelts[4,7], Paul J. Converse[8], Sandeep Tyagi[8], Sina Gerbach[9], Luzia Gyr[10], Ann-Kathrin Lemm[4,7], Johanna Volz[7], Alexandra Hölscher[7], Leon Gröschel[1,2], Eva-Maria Stemp[1,2], Norbert Heinrich[2,5,11], Florian Kloss[9], Eric L. Nuermberger[8], Dominik Schwudke[3,4,12], Michael Hoelscher[2,5,13], Christoph Hölscher[4,7] & Kerstin Walter[4,7,*]

‡ These authors contributed equally: Axel Treu, Julia Kokesch-Himmelreich, Franziska Marwitz

* Corresponding authors: Andreas Römpp (email: Andreas.Roempp@uni-bayreuth.de) and Kerstin Walter (email: kwalter@fz-borstel.de)

1 Bioanalytical Sciences and Food Analysis, University of Bayreuth, Bayreuth, Germany

2 Thematic Translational Unit Tuberculosis, German Center for Infection Research (DZIF), Partner Site Munich-Bayreuth, Munich, Germany

3 Division of Bioanalytical Chemistry, Research Center Borstel, Leibniz Lung Center, Borstel, Germany

4 Thematic Translational Unit Tuberculosis, German Center for Infection Research (DZIF), Partner Site Hamburg-Lübeck-Borstel-Riems, Hamburg, Germany

5 Institute of Infectious Diseases and Tropical Medicine, LMU University Hospital, University of Munich (LMU), Munich, Germany

[6] National and WHO Supranational Reference Center for Mycobacteria, Research Center Borstel, Borstel, Germany

[7] Division of Infection Immunology, Research Center Borstel, Leibniz Lung Center, Borstel, Germany

[8] Center for Tuberculosis Research, Division of Infectious Diseases, Johns Hopkins University School of Medicine, Baltimore, Maryland, USA

[9] Transfer Group Anti-infectives, Leibniz Institute for Natural Product Research and Infection Biology, Leibniz HKI, Jena, Germany

[10] Robotic-assisted Discovery of Antiinfectives, Leibniz Institute for Natural Product Research and Infection Biology, Leibniz-HKI, Jena, Germany

[11] Fraunhofer Institute for Translational Medicine and Pharmacology ITMP; Immunology, Infection and Pandemic Research, Munich, Germany

[12] German Center for Lung Research (DZL), Airway Research Center North (ARCN), Research Center Borstel, Leibniz Lung Center, Borstel, Germany

[13] Unit Global Health, Helmholtz Zentrum München, German Research Center for Environmental Health (HMGU), Neuherberg, Germany

Supplementary Table 1 Blood sampling scheme for determination of PK parameters in naïve BALB/c mice treated with microcrystalline or amorphous BTZ-043 at dose levels used in the BTZ-043 dose-fractionation study in Mtb-infected BALB/c mice.

Dose [mg/kg]	Animal No.	Blood sampling time points [h]					
		Pre-dose	0.5	1	2	4	8
2.5*	21-23	X		X		X	
	24-26		X		X		X
5*	31-33	X		X		X	
	34-36		X		X		X
50*	41-43	X		X		X	
	44-46		X		X		X
250*	51-53	X		X		X	
	54; 56 ***		X		X		X
250**	11-13	X		X		X	
	14-16		X		X		X

* amorphous; ** microcrystalline; *** only two animals available for sampling

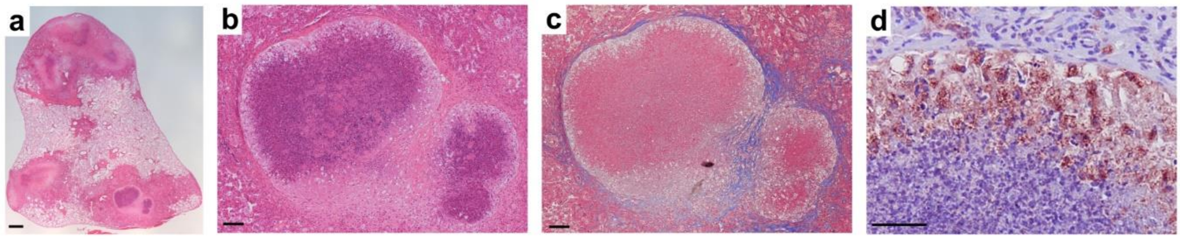

Supplementary Figure 1 Histological characterization of pulmonary lesions of Mtb-infected IL-13^{tg} mice before the start of treatment. Mice were infected with 263 CFU of Mtb H37Rv via the aerosol route. After 9 weeks, sections from formalin-fixed and paraffin-embedded lungs were prepared and subjected to histopathological and immunohistochemical analysis. **a** HE-stained specimens revealed multiple lesion types including centrally necrotizing granulomas. **b** Higher magnification image of **a** illustrating adjacent centrally necrotizing granulomas which are surrounded by a fibrous cuff detected by trichrome staining **c** and a rim of macrophages detected by CD68 staining (ab125212, Abcam, Cambridge, UK; ZytoChem-Plus kit anti-rabbit and Permanent AEC kit, both Zytomed Systems GmbH, Berlin, Germany) **d**. Representative photomicrographs of 4 mice are shown. Scale bars: a 500 µm; b & c 100 µm; d 20 µm.

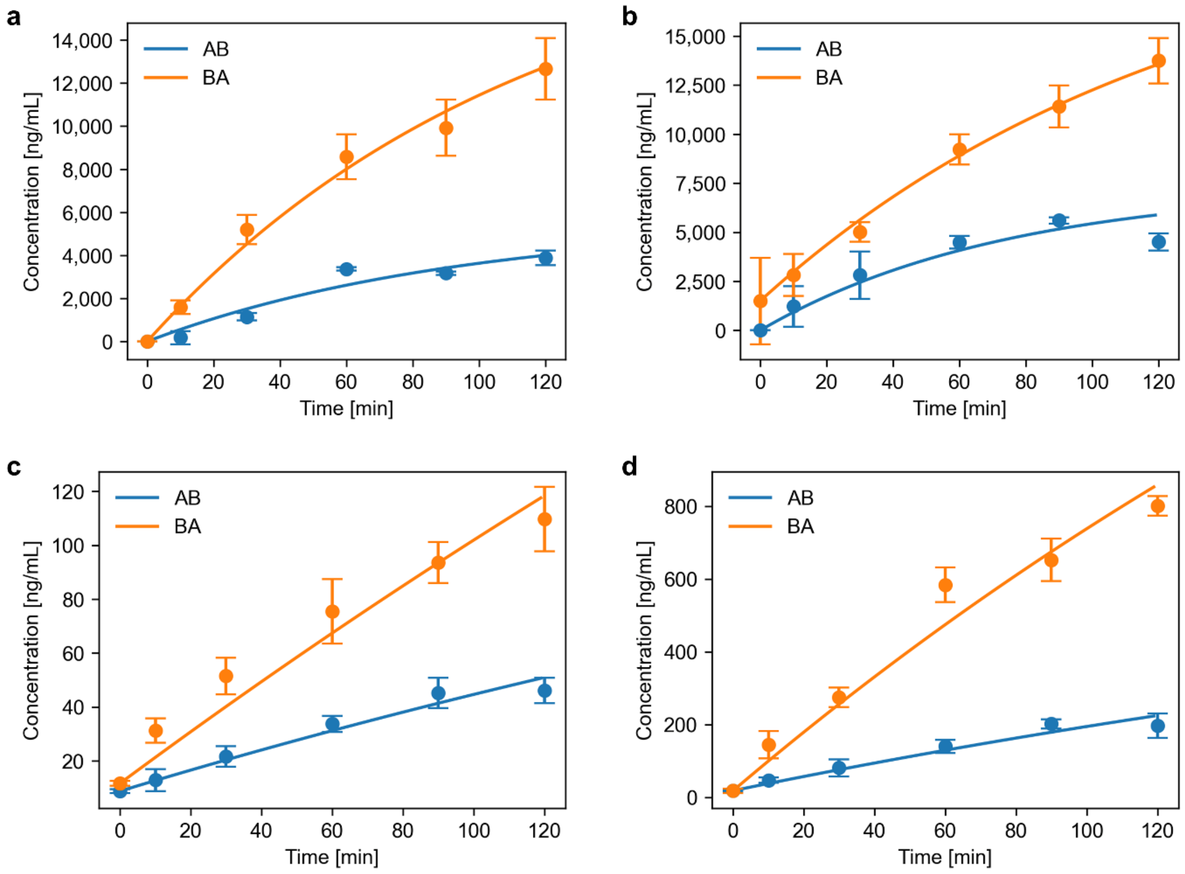

Supplementary Figure 2 *In vitro* Caco-2 permeability assay for PZA and BTZ-043. After setting up the Caco-2 assay, we first analyzed the permeability coefficients (P_{app}) of PZA as these were previously published[1]. Determination of permeability for PZA and BTZ-043 at two different concentrations (PZA: 25 and 40 µg/mL shown in **a** and **b**, respectively; BTZ-043: 1 and 4 µg/mL shown in **c** and **d**, respectively) and in both directions (AB = direction from apical to basolateral, BA = direction from basolateral to apical) in a Caco-2 monolayer. Data are shown as mean and SD (n=3). Lines represent the best fit to the data of the three replicates applying the "non-sink" condition, as reported by Tavelin *et al*.[2]. The calculated P_{app} values are listed in Supplementary Table 2. Source data are provided as Source Data file and Supplementary Data 5.

Supplementary Table 2 Calculation of P_{app} and efflux ratios for PZA and BTZ-043. Determination of permeability for PZA and BTZ-043 at two different concentrations and in both directions (AB = direction from apical to basolateral, BA = direction from basolateral to apical) in a Caco-2 monolayer. P_{app} was calculated by nonlinear curve fitting in the "non-sink" condition, as reported by Tavelin *et al*.[2]. The mean of P_{app} (n=3) was calculated from individual fitting of the three biological replicates and the corresponding standard deviation is given in the “SD” column. The efflux ratios (P_{app}(BA)/P_{app}(AB)) were determined according to Hubatsch *et al.*[3]. The determined P_{app} values for PZA 49.74 – 61.01×10^{-6} cm/s (AB) and 35.04 – 39.16×10^{-6} cm/s (BA) indicate that PZA is a highly permeable drug and confirm previously published data (70.4×10^{-6} cm/s AB)[1].

Compound	Concentration [µg/mL]	Direction	Mean P_{app} [10^{-6} cm/s]	SD	Efflux ratio
PZA	25	AB	49.74	9.25	0.79
PZA	25	BA	39.16	4.95	
PZA	40	AB	61.01	7.02	0.57
PZA	40	BA	35.04	8.73	
BTZ-043	1	AB	9.44	0.99	0.73
BTZ-043	1	BA	6.90	1.49	
BTZ-043	4	AB	11.28	1.49	1.14
BTZ-043	4	BA	12.91	1.41	

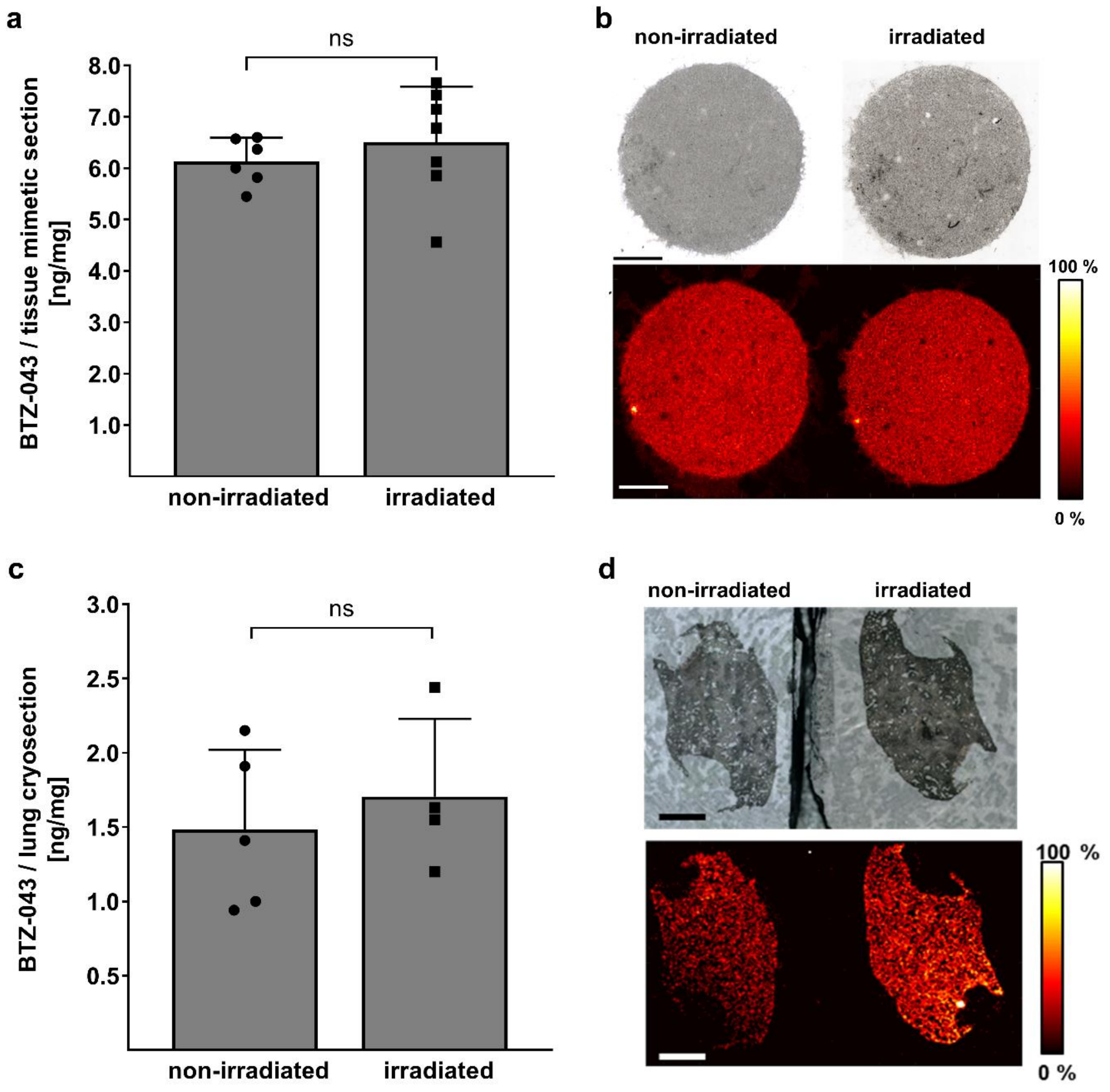

Supplementary Figure 3 Impact of γ-irradiation on BTZ-043 concentration and distribution. Serial cryosections were prepared from BTZ-043 spiked liver mimetic tissue (**a** and **b**) or from lung tissue of BTZ-043 treated mice (**c** and **d**). For both tissue types every other cryosection was irradiated with a dosage of 5.85 kGy and the impact of γ-irradiation on BTZ-043 stability was determined by LC-MS/MS (**a** and **c**) and MALDI imaging (**b** and **d**). **a** Quantification of BTZ-043 by LC-MS/MS in non-irradiated and irradiated liver mimetic cryosections. Data are shown as mean and SD (*n*=6 for non-irradiated cryosections, *n*=7 for irradiated cryosections). Concentrations were tested for normality and analyzed by a two-tailed unpaired t-test. The rout method (Q=0.1%) identified one outlier with a concentration of 24.95 ng/mg, which was removed. **b** MALDI imaging of directly neighboring either non-irradiated or irradiated liver mimetic cryosections. Pre-measurement optical image of the non-irradiated and irradiated sections (upper panel). Distribution of BTZ-043 $[M+H]^+$, $C_{17}H_{17}F_3N_3O_5S^+$, *m/z* 432.08355

(lower panel) normalized to the matrix cluster $C_{34}H_{37}N_4^+$ *m/z* 501.30127 ([2M+H]$^+$]) BTZ-043 is homogenously distributed over one section. The signal abundance is very similar in both sections (representative of 3 independent measurements). Scale bars: 2 mm.**c** Quantification of BTZ-043 by LC-MS/MS in non-irradiated and irradiated lung cryosections. Data are shown as mean and SD (*n*=4 for irradiated cryosections, *n*=5 for non-irradiated cryosections). Concentrations were tested for normality and analyzed by a two-tailed unpaired t-test. The rout method (Q=0.1%) identified one outlier with a concentration of 13.81 ng/mg, which was removed. **d** MALDI imaging of directly neighboring either non-irradiated or irradiated lung sections. Pre-measurement optical image of the non-irradiated and irradiated sections mounted on the same sample holder (upper panel). Distribution of BTZ-043 [M+H]$^+$, $C_{17}H_{17}F_3N_3O_5S^+$, *m/z* 432.08355 normalized to the matrix cluster $C_{34}H_{37}N_4^+$ *m/z* 501.30127 ([2M+H]$^+$) (lower panel). The difference in signal abundance is due to height differences on the shared sample holder. Scale bars: 1 mm. Source data are provided as Source Data file and Supplementary Data 6, 7 and 8.

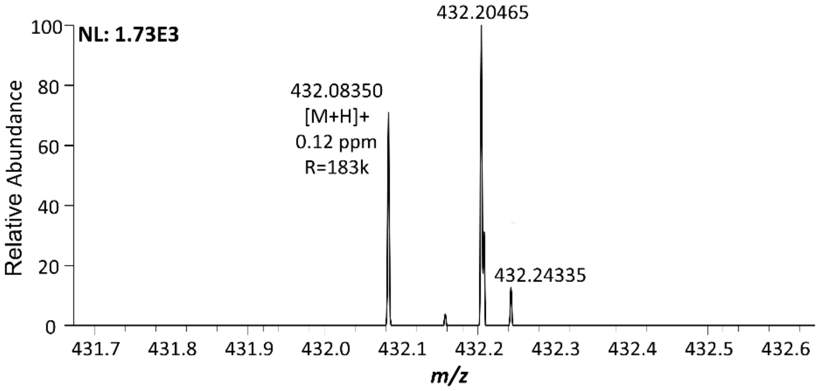

Supplementary Figure 4 Single pixel mass spectrum showing the protonated molecule of BTZ-043. The mass spectrum was extracted from the measurement shown in Figure 4. Signal to noise ratio (S/N) for the protonated molecule of BTZ-043 is 32.

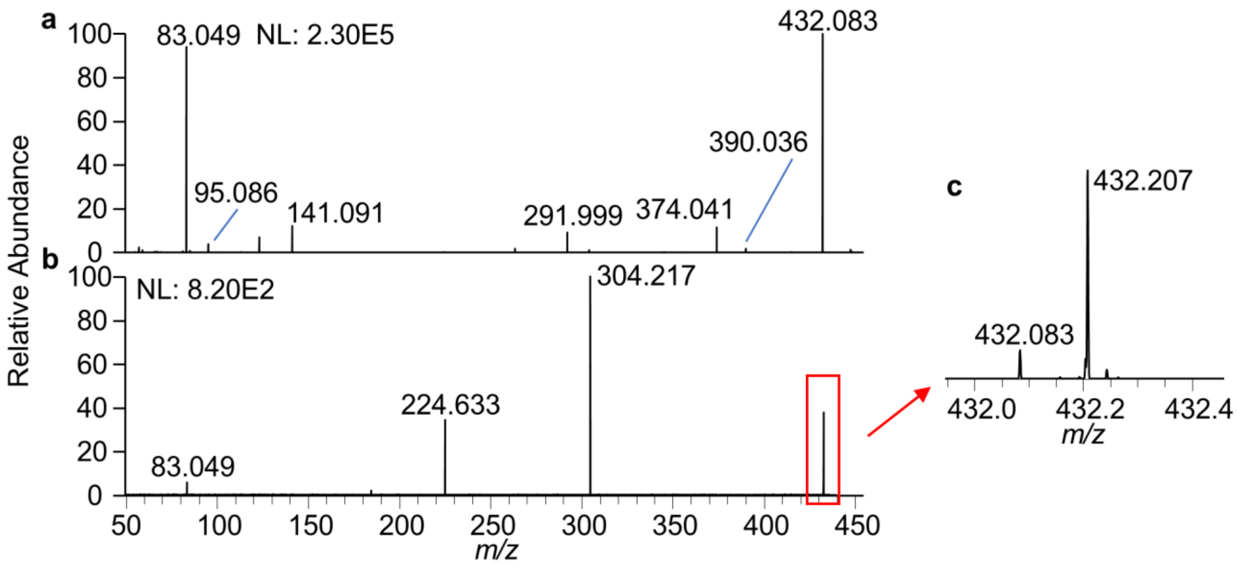

Supplementary Figure 5 MS/MS identification of BTZ-043. **a** MS/MS (HCD 26) spectrum of the protonated BTZ-043 molecule from a standard (S/N 876). **b** MS/MS spectrum of the protonated BTZ-043 molecule recorded on tissue. The on tissue MS/MS spectrum shows a number of additional peaks not present on the MS/MS spectrum of the BTZ-043 standard due to the co-isolation of neighboring peaks in the precursor isolation window (± 0.2 *m/z*). The main fragment of BTZ-043 at *m/z* 83.049 is however present in both spectra (S/N 6.2). **c** Enlargement of the on-tissue MS/MS showing the protonated molecule of BTZ-043 and a co-isolated neighboring peak.

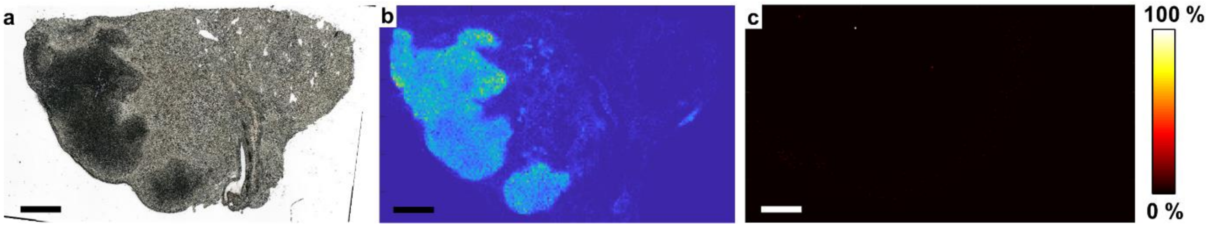

Supplementary Figure 6 Negative control for BTZ-043 conducted on an irradiated section from an Mtb-infected IL-13^{tg} mouse, which did not receive BTZ-043. **a** Pre-measurement optical image. **b** The co-detected ion *m/z* 482.36050 was used to demarcate granuloma areas. **c** Ion image of the protonated molecule of BTZ-043, $C_{17}H_{17}F_3N_3O_5S^+$, *m/z* 432.08355. Scale bars: 1 mm. Source data are provided as Supplementary Data 8.

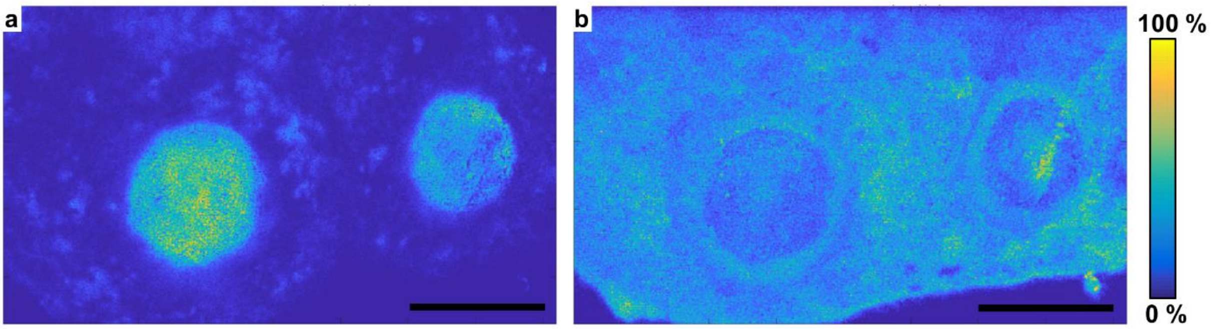

Supplementary Figure 7 Distribution of the co-detected ions used for calculating the BTZ-043 penetration in Figure 4. **a** Distribution of *m/z* 482.36050 used to demarcate the granuloma areas. **b** Distribution of *m/z* 488.44620 which was used to mark the surrounding tissue. Scale bars: 1 mm. Source data are provided as Supplementary Data 8.

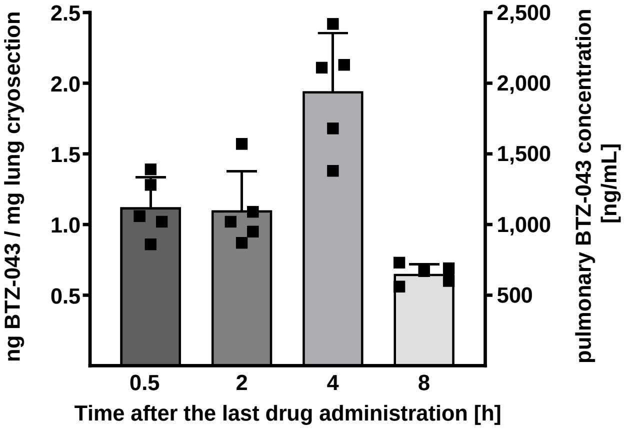

Supplementary Figure 8 Biological replicates of the pulmonary BTZ-043 concentrations shown in Figure 6a. Lung cryosections (*n*=5) obtained from an additional mouse at each time point after drug administration were analyzed. IL-13^{tg} mice were infected with 263 CFU Mtb H37Rv and after 9 weeks, animals were treated with 250 mg/kg/day of BTZ-043 for 10 days prior to lung tissue collection at indicated time points after the last administration. Serial cryosections were prepared and irradiated for following BTZ-043 quantification by LC-MS/MS. Source data are provided as Source Data file and Supplementary Data 3.

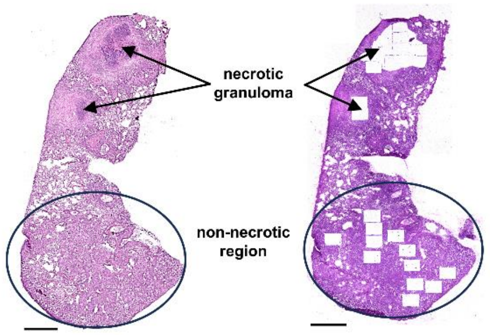

Supplementary Figure 9 Visual representation of areas collected by LCM for LC-MS/MS analysis in Figure 6b. An HE-stained lung section image is included for reference (left panel), and areas sampled by LCM are shown (right panel). Scale bars: 1mm.

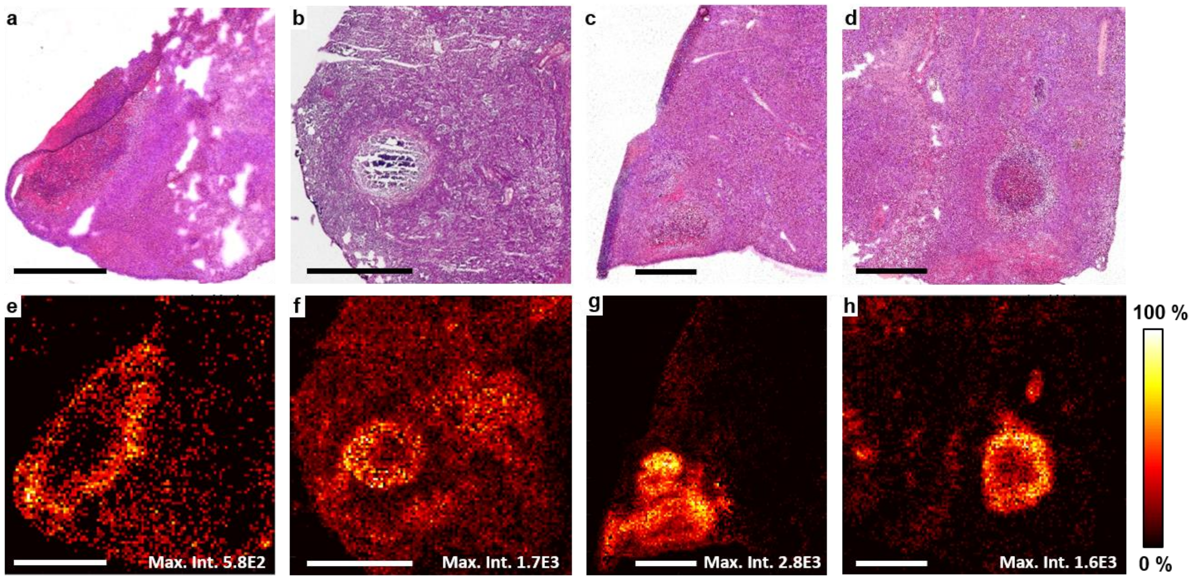

Supplementary Figure 10 Biological replicates of the MALDI imaging timeline shown in Figure 7. Mice were infected with 263 CFU Mtb H37Rv and treatment with 250mg/kg/day of BTZ043 for 10 days started 9 weeks after infection. Lung tissue was collected 0.5 h (a, e), 2 h (b, f), 4 h (c, g) and 8 h (d, h) after the last administration. **a-d** Post-measurement HE staining. **e-h** Distribution of BTZ043 $[M+H]^+$, $C_{17}H_{17}F_3N_3O_5S^+$, *m/z* 432.08355, 30 x 30 µm step size. Maximum intensities are given at the bottom of each image. Histological images are representative of 2 biological replicates. Scale bars: 1 mm. Source data are provided as Supplementary Data 2 and 8.

Supplementary Table 3 Treatment scheme used in the BTZ-043 dose-fractionation study in Mtb-infected BALB/c mice. Total daily doses were either administered in a single dose (QD) or divided into two doses (BID) which were separated by 7 - 8 h.

Treatment [mg/kg]			Total daily dose [mg/kg]	Time point and number of mice analyzed		
				day -20	day 0	week 6
Untreated			none	3	3	3
INH	10	QD	10			4
BTZ-043**	2.5	QD	2.5			4
BTZ-043**	2.5	BID	5			4
BTZ-043**	5	QD				4
BTZ-043**	5	BID	10			4
BTZ-043**	10	QD				3
BTZ-043**	10	BID	20			4
BTZ-043**	25	QD	25			4
BTZ-043**	25	BID	50			4
BTZ-043**	50	QD				4
BTZ-043**	50	BID	100			4
BTZ-043**	100	QD				4
BTZ-043*	250	QD	250			4

* microcrystalline; ** amorphous

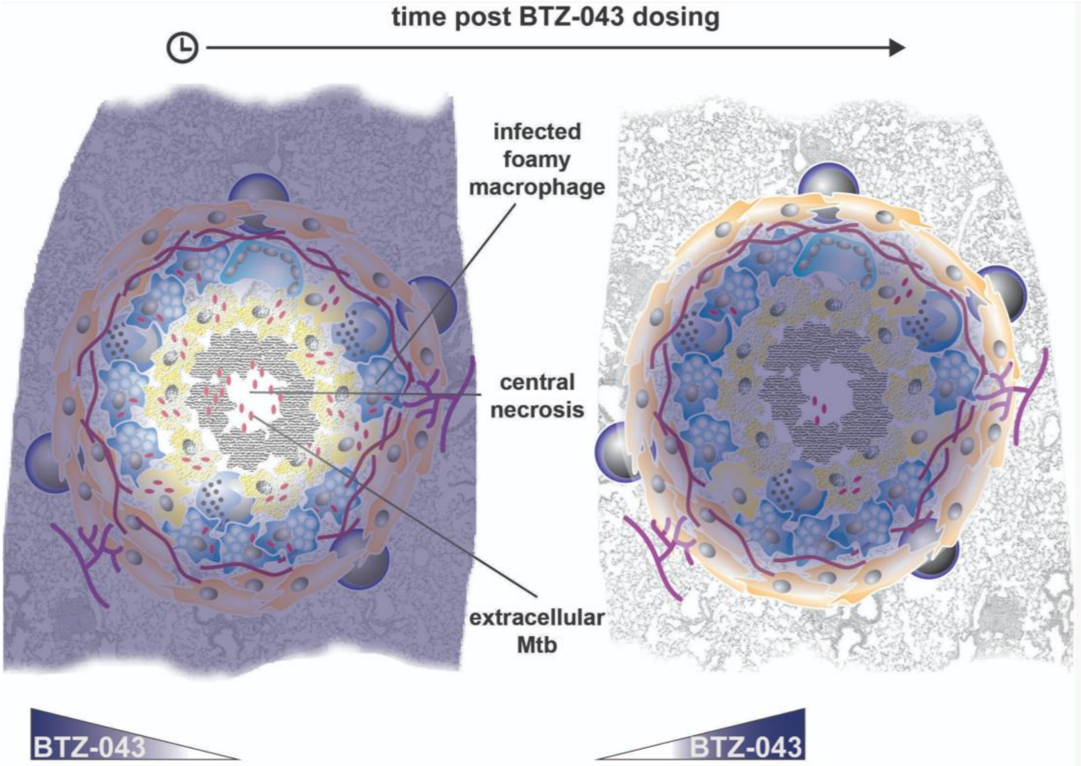

Supplementary Figure 11 Schematic illustration of BTZ-043 distribution in necrotic granulomas at different time points after administration.

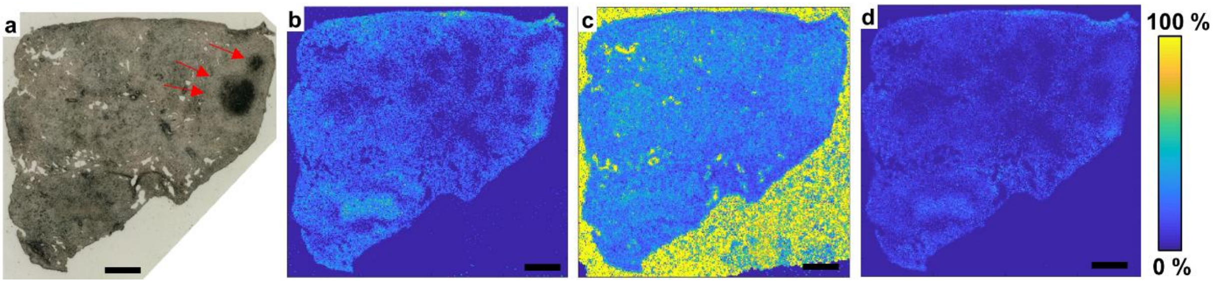

Supplementary Figure 12 Assessment of possible ion suppression effects on the distribution of BTZ-043. MALDI imaging of BTZ-043 using normalization with a deuterated standard. **a** Optical image. Note the granulomas marked by the red arrows in the top right corner **b** Distribution of BTZ-043 $[M+H]^+$, $C_{17}H_{17}F_3N_3O_5S^+$, *m/z* 432.08355 without normalization. **c** Distribution of the deuterated standard BTZ-043 D_4 $C_{17}H_{13}D_4F_3N_3O_5S^+$, *m/z* 436.10866 which was sprayed onto the section prior to the application of the matrix. **d** Distribution of BTZ-043 normalized to the signal of the deuterated standard. As there is no apparent difference between the non-normalized and normalized distribution of BTZ-043, especially in the granuloma areas, a possible influence of ion suppression on the observed distribution can be ruled out. Scale bars: 1 mm. Source data are provided as Supplementary Data 8.

Supplementary Table 4 Calculated root mean square error (RMSE) of the BTZ-043 *m*/*z* ratio in the imaging measurements included in this study. The figure of the corresponding MS image is given in the first column. RMSE values and corresponding numbers of spectra where the protonated molecule of BTZ043, ($[M+H]^+$,$C_{17}H_{17}F_3N_3O_5S^+$, *m/z* 432.08355) was detected are given in the last two columns.

Figure	RMSE [ppm]	Number of spectra
5e	0.66	53188
7e	0.56	11013
7f	0.36	11379
7g	0.86	18827
7h	0.65	4459
S3b	0.54	27697
S3d	0.64	9074
S9e	0.77	2336
S9f	0.72	21368
S9g	0.73	6011
S9h	1.11	4935
S10b	0.48	32218

Supplementary method

Quantification of drugs for Caco-2 assay

BTZ-043 and PZA samples were precipitated with MeOH (1:4) or ACN (1:20), respectively. All samples were analyzed using a Vanquish Horizon UHPLC system (Thermo Fisher Scientific, Bremen, Germany) coupled to a Thermo Scientific QExactive HF-X Orbitrap mass spectrometer (Thermo Fisher Scientific, Bremen, Germany) using a heated electrospray ionization (HESI) source. The HESI source was set to 3.5 kV, capillary temperature 320 °C, funnel RF level 40, sheath gas pressure 49, auxiliary gas 10, auxiliary gas heater temperature 300 °C. The automatic gain control (AGC) target was set to 10E+6 and the injection time was set to 200 ms. An m/z range of 150 to 1,800 and 80 to 1,000 was selected for BTZ-043 and PZA, respectively. Mass spectra were acquired at a resolution of 60,000 full width at half maximum and at positive mode. For the liquid chromatography separation, a reverse-phase column (Acquity UHPLC, HSS T3, 50 x 2.1 mm, 1.8 µM, 100 Å, Waters, Milford, USA) was used for BTZ-043 and HILIC column (SeQuant® ZIC®-HILIC, 150 x 2.1 mm, 3.5 µM, 200 Å, Merck Millipore, Darmstadt, Germany) was chosen for PZA. For both analyses, eluent A was water containing 0.1% FA and eluent B was ACN containing 0.1% FA. The gradient for the reverse-phase column was used with a flow rate of 0.6 $mL·min^{-1}$ and with the following gradient: gradient of eluent B from 5% to 98% in 4 min, isocratic for 2.5 min, gradient to 5% in 0.5 min and equilibration for 2 min at 5% of eluent B. The HILIC column was operated using an isocratic flow of 97% eluent B (0.4 $mL·min^{-1}$) for 1 min followed by a gradient to 50% within 2 min, an isocratic hold for 3 min, a gradient to 97% within 1 min and an equilibration phase of 8 min. For both columns the temperature was set to 25 °C and the autosampler was set to 4 °C. The injection volume was 5 µL. Integration of the peak areas of BTZ-043 and PZA were

done using TraceFinder 4.1 SP3. The data were further analyzed with Python. The permeability coefficient (P_{app}), and efflux ratio were calculated according to Hubatsch et al. [3].

Assessment of impact of γ-irradiation on BTZ-043 stability

Preparation of lung tissue from BTZ-043 treated mice. Naïve BALB/c mice were treated with a dose of 250 mg/kg/day BTZ-043 by oral gavage for 5 consecutive days. Mice were sacrificed 1 h after the last administration, individual lung lobes were isolated, snap frozen in liquid nitrogen and stored at -80 °C until cryosectioning.

Preparation of mimetic tissue model. For the preparation of the mimetic tissue model[4] a previously described protocol[5] was slightly modified. Briefly, tissue homogenate was prepared by placing 1 g of portioned mouse liver tissue in a steel ball Precellys tube (2 mL, Precellys Lysing kit, Bertin Technologies, Montigny- le-Bretonneux, France) and using the FastPrep 24 bead homogenizer (MP Biomedicals, Solon, OH, USA) without the addition of solvent. Based on the determined mass of the liver a defined volume of BTZ-043 solution [1.6 mg/mL solved in ACN] was spiked into the homogenate to yield a concentration which is similar to the pulmonary BTZ-043 concentration of drug treated mice (9.7 µg/g; drug/homogenate). The spiked homogenate was mixed using the FastPrep 24 bead homogenizer to ensure an even distribution of BTZ-043 throughout the homogenate. A mold was prepared from a 2 mL syringe which was pre-cooled on dry ice before the spiked homogenate was added to the mold. Once the homogenate was completely frozen the syringe was removed and the tissue stick was stored at -80 °C until cryosectioning.

Cryosectioning and γ-irradiation. Serial cryosections (12 µm) from frozen lung tissue and frozen mimetic tissue model were cut using a Leica CM3050s cryostat (Leica

Microsystem, Wetzlar, Germany). Sections were thaw mounted onto adhesive glass slides (SuperFrost™, Langenbrinck, Emmendingen, Germany) and stored at -80 °C until further processing. To assess the impact of γ-irradiation on BTZ-043 stability our previously described protocol[6] was used. Briefly, every other lung cryosection and every other liver mimetic cryosection was irradiated on dry ice with a dose of 5.85 kGy (BIOBEAM 8000, Gamma-Service Medical GmbH, Leipzig, Germany) while directly neighboring sections were processed identically except for the irradiation process and served as controls. Cryosections were stored at -80 °C until subsequent analyses.

LC-MS/MS of cryosections. Sample preparation and quantification of BTZ-043 in irradiated or non-irradiated pulmonary tissue cryosections was according to the methods of the main paper. The following modifications were made for sample preparation and quantification of BTZ-043 in irradiated or non-irradiated liver mimetic cryosections. Chromatography was performed with an Agilent 1100 Series HPLC (Agilent Technologies, Santa Clara, CA, USA) using a XSelect® CSH™ C18 column (2.1 inner diameter x 150 mm length with 5 µm particle size, pore-size 130 Å) (Waters, Corporation, Milford, MA, USA). Column temperature was set to 30 °C. The gradient with mobile phase A (1% FA) and B (ACN) was as follows: 90% A and 10% B for 1 minute, followed by a stepwise increase of B to 50% at 2 min, 80% at 5 min and 90% at 6 min. This was kept isocratic until 10 min and subsequently returned to 10% B within 1 min. These conditions were kept from min 11 to 14. For analysis the triple quadrupole mass spectrometer XEVO TQ-MS (Waters, Corporation, Milford, MA, USA) with an ESI source was operated in positive ion mode. The measured *m/z*-transitions for BTZ-043 and reserpine as well as capillary voltage, cone gas flow and desolvation gas flow were the same as in the main paper. For BTZ-043 the *m/z*-transition 432.1 → 83.1 was used as quantifier. Source temperature and desolvation

temperature were 150 °C and 350 °C, respectively. For BTZ-043 a cone voltage of 30 V, a collision energy of 30 eV and a dwell time of 0.2 s were used. For reserpine the respective values were 45 V, 50 eV and 0.1 s.

For calibration curve preparation liver homogenate from naïve mice was extracted and diluted with 80% ACN/20% FA (1%), containing reserpine (20 ng/mL per sample) to the final solution containing about 2.8 µg tissue/mL, which equals the average weight of the cryosections that were extracted for analysis. This solvent was spiked with BTZ-043 for a calibration curve ranging from 0.001-0.1 µg/mL.

MALDI imaging of liver mimetic cryosections. Sample preparation for MALDI imaging of mimetic tissue was carried out as for the lung sections described in the main paper. The measurements were conducted in the mass range *m/z* 420-510 with a pixel size of 75 x 75 µm. Ion images and RGB overlays were generated in MSiReader Version 1.0[7] with a bin width of 2 ppm after conversion of the Thermo RAW files to the imzML format[8] (imzML Converter Version 2.0.4[9]).

References

1 Lakshminarayana, S. B. *et al.* Comprehensive physicochemical, pharmacokinetic and activity profiling of anti-TB agents. *J. Antimicrob. Chemother.* **70**, 857-867 (2015).

2 Tavelin, S., Gråsjö, J., Taipalensuu, J., Ocklind, G. & Artursson, P. Applications of epithelial cell culture in studies of drug transport. *Methods Mol. Biol.* **188**, 233-272 (2002).

3 Hubatsch, I., Ragnarsson, E. G. & Artursson, P. Determination of drug permeability and prediction of drug absorption in Caco-2 monolayers. *Nat. Protoc.* **2**, 2111-2119 (2007).

4 Groseclose, M. R. & Castellino, S. A mimetic tissue model for the quantification of drug distributions by MALDI imaging mass spectrometry. *Anal. Chem.* **85**, 10099-10106 (2013). https://doi.org:10.1021/ac400892z

5 Barry, J. A., Groseclose, M. R. & Castellino, S. Quantification and assessment of detection capability in imaging mass spectrometry using a revised mimetic tissue model. *Bioanalysis* **11**, 1099-1116 (2019). https://doi.org:10.4155/bio-2019-0035

6 Walter, K. *et al.* Interleukin-13-Overexpressing Mice Represent an Advanced Preclinical Model for Detecting the Distribution of Antimycobacterial Drugs within Centrally Necrotizing Granulomas. *Antimicrob Agents Chemother* **66**, e0158821 (2022).

7 Bokhart, M. T., Nazari, M., Garrard, K. P. & Muddiman, D. C. MSiReader v1.0: Evolving Open-Source Mass Spectrometry Imaging Software for Targeted and Untargeted Analyses. *J Am Soc Mass Spectrom* **29**, 8-16 (2018).

8 Schramm, T. *et al.* imzML--a common data format for the flexible exchange and processing of mass spectrometry imaging data. *J. Proteomics* **75**, 5106-5110 (2012). https://doi.org:10.1016/j.jprot.2012.07.026

9 Race, A. M., Styles, I. B. & Bunch, J. Inclusive sharing of mass spectrometry imaging data requires a converter for all. *J. Proteomics* **75**, 5111-5112 (2012). https://doi.org:10.1016/j.jprot.2012.05.035
