# Supplementary material for: The clinical-stage drug BTZ-043 accumulates in murine tuberculosis lesions and efficiently acts against Mycobacterium tuberculosis
Source: Nat Commun. 2025 Jan 18;16:826. doi: 10.1038/s41467-025-56146-9 (PMC11742723; doi:10.1038/s41467-025-56146-9)
Supplement: Supplementary file 3 — Description of Additional Supplementary Files [file 41467_2025_56146_MOESM3_ESM.pdf]

Description of Additional Supplementary Files

Supplementary Data 1

Title: LC-MS/MS measurements of BTZ-043 in mouse plasma

Description: Calculation of concentration-time curves for pharmacokinetic profile of BTZ-043 in mice. Data shown in Figure 2.

Supplementary Data 2

Title: MALDI MS imaging of BTZ-043 in mouse tissue sections

Description: MALDI ion images have been cropped in some cases in order to facilitate comparison between different granulomas in Figure 7 and Supplementary Figure 10. Acomparis on between cropped and complete measurement is shown here.

Supplementary Data 3

Title: LC-MS/MS measurements of BTZ-043 in lung cryosections

Description: Data processing pipeline until the determined BTZ-043 quantities for Figure 6a and Supplementary Figure 8.

Supplementary Data 4

Title: LC-MS/MS measurements of BTZ-043 in necrotic granulomas and non-necrotic tissue collected by laser-capture microdissection

Description: Data processing pipeline until the determined BTZ-043 quantities for Figure 6b.

Supplementary Data 5

Title: LC-MS/MS measurements of BTZ-043 and PZA in cell culture

Description: Calculation of drug compound concentrations in apical and basal compartment for permeability assay. Data shown in Supplementary Figure 2.

Supplementary Data 6

Title: LC-MS/MS measurements of BTZ-043 in irradiated and non-irradiated liver mimetic cryosections

Description: Data processing pipeline until the determined BTZ-043 quantities for Supplementary Figure 3a.

Supplementary Data 7

Title: LC-MS/MS measurements of BTZ-043 in irradiated and non-irradiated lung cryosections

Description: Data processing pipeline until the determined BTZ-043 quantities for Supplementary Figure 3c.

Supplementary Data 8

Title: MALDI MS imaging of mouse tissue sections

Description: Intensity values along with pixel coordinates for all MALDI MS images shown in this study.
