# Supplementary material for: The clinical-stage drug BTZ-043 accumulates in murine tuberculosis lesions and efficiently acts against Mycobacterium tuberculosis
Source: Nat Commun. 2025 Jan 18;16:826. doi: 10.1038/s41467-025-56146-9 (PMC11742723; doi:10.1038/s41467-025-56146-9)
Supplement: Supplementary file 12 — Reporting Summary [file 41467_2025_56146_MOESM12_ESM.pdf]

nature portfolio

Corresponding author(s): Kerstin Walter
Andreas Römpp

Last updated by author(s): Dec 20, 2024

Reporting Summary

Nature Portfolio wishes to improve the reproducibility of the work that we publish. This form provides structure for consistency and transparency in reporting. For further information on Nature Portfolio policies, see our Editorial Policies and the Editorial Policy Checklist.

Statistics

For all statistical analyses, confirm that the following items are present in the figure legend, table legend, main text, or Methods section.

n/a	Confirmed	
☐	☒	The exact sample size (*n*) for each experimental group/condition, given as a discrete number and unit of measurement
☐	☒	A statement on whether measurements were taken from distinct samples or whether the same sample was measured repeatedly
☐	☒	The statistical test(s) used AND whether they are one- or two-sided<br>*Only common tests should be described solely by name; describe more complex techniques in the Methods section.*
☒	☐	A description of all covariates tested
☐	☒	A description of any assumptions or corrections, such as tests of normality and adjustment for multiple comparisons
☐	☒	A full description of the statistical parameters including central tendency (e.g. means) or other basic estimates (e.g. regression coefficient) AND variation (e.g. standard deviation) or associated estimates of uncertainty (e.g. confidence intervals)
☐	☒	For null hypothesis testing, the test statistic (e.g. *F*, *t*, *r*) with confidence intervals, effect sizes, degrees of freedom and *P* value noted<br>*Give P values as exact values whenever suitable.*
☒	☐	For Bayesian analysis, information on the choice of priors and Markov chain Monte Carlo settings
☒	☐	For hierarchical and complex designs, identification of the appropriate level for tests and full reporting of outcomes
☒	☐	Estimates of effect sizes (e.g. Cohen's *d*, Pearson's *r*), indicating how they were calculated

Our web collection on statistics for biologists contains articles on many of the points above.

Software and code

Policy information about availability of computer code

Data collection	3500 Series Data Collection Software 3; Build id 3500v.3.3<br>cellSens imaging software<br>NIS-Elements software<br>Rotor-Gene Q-Rex or LightCycler 480 software version LCS480 1.5.1.62<br>MassLynx 4.1<br>Thermo software Q-Eactive- HF tune Version 2.9<br>TransMIT software Master Control Program Version 3.9
Data analysis	Sequencing Analysis Software 7.0 Build id B01<br>TargetLynx<br>Xcalibur 4.0 from Thermo Fisher Scientific<br>MSiReader Version 1.0<br>imzML Converter Version 2.0.4<br>Penetration analysis tool v0.3<br>Microsoft Excel 365<br>GraphPad Prism 9<br>TraceFinder 4.1 SP3

For manuscripts utilizing custom algorithms or software that are central to the research but not yet described in published literature, software must be made available to editors and reviewers. We strongly encourage code deposition in a community repository (e.g. GitHub). See the Nature Portfolio guidelines for submitting code & software for further information.

Data

Policy information about availability of data

All manuscripts must include a data availability statement. This statement should provide the following information, where applicable:

- Accession codes, unique identifiers, or web links for publicly available datasets
- A description of any restrictions on data availability
- For clinical datasets or third party data, please ensure that the statement adheres to our policy

Source data and supplementary data are available within the manuscript for all applicable figures. Raw data for MS in tissue sections has been uploaded at Zenodo (HPLC-MS/MS) and Metaspace (MALDI imaging).

Research involving human participants, their data, or biological material

Policy information about studies with human participants or human data. See also policy information about sex, gender (identity/presentation), and sexual orientation and race, ethnicity and racism.

Reporting on sex and gender	*Use the terms sex (biological attribute) and gender (shaped by social and cultural circumstances) carefully in order to avoid confusing both terms. Indicate if findings apply to only one sex or gender; describe whether sex and gender were considered in study design; whether sex and/or gender was determined based on self-reporting or assigned and methods used. Provide in the source data disaggregated sex and gender data, where this information has been collected, and if consent has been obtained for sharing of individual-level data; provide overall numbers in this Reporting Summary. Please state if this information has not been collected. Report sex- and gender-based analyses where performed, justify reasons for lack of sex- and gender-based analysis.*
Reporting on race, ethnicity, or other socially relevant groupings	*Please specify the socially constructed or socially relevant categorization variable(s) used in your manuscript and explain why they were used. Please note that such variables should not be used as proxies for other socially constructed/relevant variables (for example, race or ethnicity should not be used as a proxy for socioeconomic status). Provide clear definitions of the relevant terms used, how they were provided (by the participants/respondents, the researchers, or third parties), and the method(s) used to classify people into the different categories (e.g. self-report, census or administrative data, social media data, etc.) Please provide details about how you controlled for confounding variables in your analyses.*
Population characteristics	*Describe the covariate-relevant population characteristics of the human research participants (e.g. age, genotypic information, past and current diagnosis and treatment categories). If you filled out the behavioural & social sciences study design questions and have nothing to add here, write "See above."*
Recruitment	*Describe how participants were recruited. Outline any potential self-selection bias or other biases that may be present and how these are likely to impact results.*
Ethics oversight	*Identify the organization(s) that approved the study protocol.*

Note that full information on the approval of the study protocol must also be provided in the manuscript.

Field-specific reporting

Please select the one below that is the best fit for your research. If you are not sure, read the appropriate sections before making your selection.

☒ Life sciences ☐ Behavioural & social sciences ☐ Ecological, evolutionary & environmental sciences

For a reference copy of the document with all sections, see nature.com/documents/nr-reporting-summary-flat.pdf

Life sciences study design

All studies must disclose on these points even when the disclosure is negative.

Sample size	Sample size for mouse studies was chosen based on own experience with similar models and published data showing similar experiments.
Data exclusions	In Figure 1a and 1b one lung isolate (50mg/kg treatment for 8 weeks) was not evaluable due to contamination of agar plates. In Supplementary Figure 3 two samples were excluded. In Supplementary Fig. 3a the rout method (Q=0.1%) identified one outlier (24.95 ng/mg) which was removed and in Supplementary Fig. 3c the rout method (Q=0.1%) identified one outlier (13.81 ng/mg) which was removed. This information is also given in the legend of Supplementary Figure 3.
Replication	The number of biological replicates for each experiment is indicated in the Figure and Table legends.
Randomization	For BALB/c studies, female mice were purchased and delivered as a single batch per experiment and mice were randomly assigned into treatment groups prior to infection. In order to avoid an imbalance regarding the health status of IL-13tg mice, animals with the same health status were distributed evenly between the treatment or non-treatment group before the start of therapy.

Blinding	Overall, the experiments were performed in a non-blinded fashion except for histological examination shown in Fig 3b which was performed by a scientist blinded to the treatment group.

Reporting for specific materials, systems and methods

We require information from authors about some types of materials, experimental systems and methods used in many studies. Here, indicate whether each material, system or method listed is relevant to your study. If you are not sure if a list item applies to your research, read the appropriate section before selecting a response.

Materials & experimental systems

n/a	Involved in the study
☐	☒ Antibodies
☐	☒ Eukaryotic cell lines
☒	☐ Palaeontology and archaeology
☐	☒ Animals and other organisms
☒	☐ Clinical data
☒	☐ Dual use research of concern
☒	☐ Plants

Methods

n/a	Involved in the study
☒	☐ ChIP-seq
☒	☐ Flow cytometry
☒	☐ MRI-based neuroimaging

Antibodies

Antibodies used	primary antibody:<br>CD68 (ab125212, Abcam, Cambridge, U.K)<br>secondary antibodies:<br>goat anti rabbit (111-065-144, Jackson Immunoresearch, Suffolk, UK)<br>ZytoChem-Plus Kit Anti-rabbit (Zytomed Systems GmbH, Berlin, Germany)
Validation	Primary antibody was titrated before analysis.

Eukaryotic cell lines

Policy information about cell lines and Sex and Gender in Research

Cell line source(s)	DSMZ no.: ACC169
Authentication	The Caco-2 cell line was directly used upon arrival from the providing company.
Mycoplasma contamination	The CaCo-2 cell line was tested negative for Mycoplasma using InvivoGen test kit.
Commonly misidentified lines (See ICLAC register)	Does not apply.

Animals and other research organisms

Policy information about studies involving animals; ARRIVE guidelines recommended for reporting animal research, and Sex and Gender in Research

Laboratory animals	This manuscript includes two mouse strains (species Mus musculus):<br>• BALB/c female mice 8-10 weeks old (JHU) or body weight around 20 g (RCB)<br>• IL-13tg (Tg(CD4-Il13)431Anjm on BALB/c genetic background) male and female mice; body weight between 18 g and 34 g before infection<br>Mice were housed socially with a maximum of 5 mice per cage. Animals had access to irradiated chow and ozone-enriched tap water ad libitum with daily visual control. Cages and bedding were changed once a week and nesting material was provided for enrichment. Twelve hours light/dark cycle were employed and mice were maintained at temperatures between 20°C and 24°C with mean relative humidity between 26% and 68%.
Wild animals	This study did not involve wild animals.
Reporting on sex	Female BALB/c mice were used for dose escalation study and dose fractionation study. Male and female IL-13tg mice were used for drug efficacy studies, drug distribution and drug concentration studies.
Field-collected samples	This study did not involve collection of field samples.
Ethics oversight	Animal experiments at the RCB were approved by the animal research ethics committee of the federal state of Schleswig-Holstein

Ethics oversight	and by the Ministry of Energy, Agriculture, the Environment, Nature, and Digitalization, Schleswig-Holstein, Kiel, Germany (approval numbers 3-1/15, 69-6/16, and 84-9/20). Animal experiments at JHU were reviewed and approved by the Johns Hopkins University Animal Care and Use Committee (protocol #MO15M479).

Note that full information on the approval of the study protocol must also be provided in the manuscript.

Plants

Seed stocks	*Report on the source of all seed stocks or other plant material used. If applicable, state the seed stock centre and catalogue number. If plant specimens were collected from the field, describe the collection location, date and sampling procedures.*
Novel plant genotypes	*Describe the methods by which all novel plant genotypes were produced. This includes those generated by transgenic approaches, gene editing, chemical/radiation-based mutagenesis and hybridization. For transgenic lines, describe the transformation method, the number of independent lines analyzed and the generation upon which experiments were performed. For gene-edited lines, describe the editor used, the endogenous sequence targeted for editing, the targeting guide RNA sequence (if applicable) and how the editor was applied.*
Authentication	*Describe any authentication procedures for each seed stock used or novel genotype generated. Describe any experiments used to assess the effect of a mutation and, where applicable, how potential secondary effects (e.g. second site T-DNA insertions, mosiacism, off-target gene editing) were examined.*
